# Functional Changes in the Snail Statocyst System Elicited by Microgravity

**DOI:** 10.1371/journal.pone.0017710

**Published:** 2011-03-29

**Authors:** Pavel M. Balaban, Aleksey Y. Malyshev, Victor N. Ierusalimsky, Nikolay Aseyev, Tania A. Korshunova, Natasha I. Bravarenko, M. S. Lemak, Matvey Roshchin, Igor S. Zakharov, Yekaterina Popova, Richard Boyle

**Affiliations:** 1 Institute of Higher Nervous Activity and Neurophysiology, Russian Academy of Sciences, Moscow, Russia; 2 Koltzov Institute of Developmental Biology, Russian Academy of Sciences, Moscow, Russia; 3 NASA Ames Research Center, Moffett Field, California, United States of America; Max-Planck-Institut für Neurobiologie, Germany

## Abstract

**Background:**

The mollusk statocyst is a mechanosensing organ detecting the animal's orientation with respect to gravity. This system has clear similarities to its vertebrate counterparts: a weight-lending mass, an epithelial layer containing small supporting cells and the large sensory hair cells, and an output eliciting compensatory body reflexes to perturbations.

**Methodology/Principal Findings:**

In terrestrial gastropod snail we studied the impact of 16- (Foton M-2) and 12-day (Foton M-3) exposure to microgravity in unmanned orbital missions on: (i) the whole animal behavior (*Helix lucorum* L.), (ii) the statoreceptor responses to tilt in an isolated neural preparation (*Helix lucorum* L.), and (iii) the differential expression of the Helix pedal peptide (HPep) and the tetrapeptide FMRFamide genes in neural structures (*Helix aspersa* L.). Experiments were performed 13–42 hours after return to Earth. Latency of body re-orientation to sudden 90° head-down pitch was significantly reduced in postflight snails indicating an enhanced negative gravitaxis response. Statoreceptor responses to tilt in postflight snails were independent of motion direction, in contrast to a directional preference observed in control animals. Positive relation between tilt velocity and firing rate was observed in both control and postflight snails, but the response magnitude was significantly larger in postflight snails indicating an enhanced sensitivity to acceleration. A significant increase in mRNA expression of the gene encoding HPep, a peptide linked to ciliary beating, in statoreceptors was observed in postflight snails; no differential expression of the gene encoding FMRFamide, a possible neurotransmission modulator, was observed.

**Conclusions/Significance:**

Upregulation of statocyst function in snails following microgravity exposure parallels that observed in vertebrates suggesting fundamental principles underlie gravi-sensing and the organism's ability to adapt to gravity changes. This simple animal model offers the possibility to describe general subcellular mechanisms of nervous system's response to conditions on Earth and in space.

## Introduction

Of all the environmental parameters under which a living organism has been exposed in the course of its evolution only gravity has remained constant. Predation, climate, vegetation, and terrestrial or aquatic habitation, for example, have changed, but the intensity and direction of gravity have not. Most extant vertebrates are gnathostomes or jawed, but in the Devonian period about 400 million years ago the majority of vertebrates were ostracoderms or jawless. Despite this shift within vertebrata, fossil evidence shows that the elaborate sensory structures used to sense the acceleration forces are remarkably conserved [1]. Although less is available in the fossil record on invertebrate sensory structures, recent gene expression studies suggest that gravisensing statocyst system in eumetazoans is in the same way elemental and highly conserved [2].

The organism's ability to detect gravity and to live under a gravitational load is critical for its survival. Even rudimentary ciliated protozoa display positive or negative geotaxis [3]. Most if not all invertebrate species can orient their bodies' axis with respect to gravity, but only in a few crustaceans, insects and mollusks has gravireception been more thoroughly studied. In insects it is remarkably robust, but equally complex in design. In gastropods the statocyst is the predominant gravisensing organ, and it has clear similarities to its vertebrate counterparts: a weight-lending mass comprised of calcium carbonate particles called statoconia (the particles are called otoconia in mammals), and an epithelial layer containing small supporting cells carrying microvilli and giant sensory hair cells carrying, in some species such as land snails, true (9+2)-type kinocilia (supporting cells and kinocilium-bearing hair cells populate the mammalian vestibular sensory regions). Differences lie in the mode of transmission to higher centers. In the snail the nerve bundle from each statocyst projects rostrally into the cerebral ganglion on each side, and each fiber is the axon of an individual hair cell. In vertebrates the nerve bundle from each otolith organ projects into the brainstem bilaterally, and each fiber is the axon of a cranial nerve afferent receiving synaptic inputs from the more numerous hair cells. Similarities once again appear in the existence of efferent fibers in the statocyst nerve projecting from other neural areas for both gastropods [4] and vertebrates [5].

With the advent of space flight it is possible to address fundamental questions on the biological principle(s) of gravireception in animals, from humans to invertebrates. A key question is: despite the constancy of gravity, does the nervous system adapt to novel transitions in gravity states, even for transitions of brief duration measured in days? Another key question is: because of the elemental nature of gravireception, do animals respond similarly to altered gravity states and adapt to the new, and in the case of postflight the original, environment? In this study we address these questions in an invertebrate model.

In two unmanned orbital missions on the Russian spacecrafts Foton M-2 and M-3 we investigated the response of the snail gravisensing system to the space mission, which persists largely of the µG exposure, within a brief delay after landing. The terrestrial snail *Helix lucorum* Linnaeus (*Pulmonata, Gastropoda*) offered an excellent model of study. Because these animals are small, we had a sufficient sample size and still remained within our volume limit. Snails are resilient and can remain metabolically active under confined conditions for weeks. Importantly, certain behaviors and the cellular networks underlying them are well described in this animal [6]. Experiments were designed to evaluate the impact of changes in gravity states on three separate but related areas of statocyst function. First, we studied the behavioral (whole animal) response to sudden orientation shifts with respect to gravity, known as negative gravitaxis. This is a ubiquitous behavior found throughout nature. Second, we used electrophysiological techniques in the same subpopulation of snails examined for their behavioral response to tilt to determine the postflight response of individual hair cells to controlled tilts. In vertebrates, the cranial nerve afferents reporting to the brain the head acceleration sensed by the inner ear hair cells show a hypersensitivity, or upregulation, of the discharge response during the first days following microgravity exposure [7]. And third, in a separate population of smaller snails (*H. aspersa*) we examined the subcellular expression patterns of specific neuropeptide genes. Primary statocyst receptor cells express a product of the preproHPep gene - a pedal peptide, HPep, under physiological load [8], and regulation of this gene expression pattern might signal how the statocyst receptor is ‘tuned’ by the gravity vector. The second neuropeptide studied was FMRFamide, an endogenous peptide found in the nervous system throughout the animal kingdom. In invertebrates FMRFamides in fact constitute a major class of peptide neurotransmitters.

Preliminary conference reports on Foton M-2 and Foton M-3 flight experiments were presented [9], [10].

## Materials and Methods

### Creating persistent conditions for microgravity

The Foton series of spacecraft uses a modified Soyuz-U launcher and a recoverable satellite. The temperature inside the satellite is controlled via heaters and fans, and the satellite is tumbled in orbit to expose the outer surface evenly to the Sun. The satellite is sealed tightly, but the atmospheric composition is not maintained. As a result only short duration missions of 2–3 weeks with species requiring minimal advanced life support are flown on this platform. Temperature measures within the satellite were down-linked periodically from orbit to control the delayed asynchronous control experiments. A temperature sensor was also placed within the snail habitat to validate its correspondence to the satellite temperature. Because the satellite in orbit is in a free fall towards Earth, the snails were exposed to µG not zero G, in the range of 10^−3^–10^−5^ G. For Foton M-2 the snails were returned to the laboratory at 30 h after landing. For M-3 the delay was significantly reduced to 13 h by chartering a private flight.

### Animals

Experimental protocol was in accordance with the USA NIH “Guide for the Care and Use of Laboratory Animals” and approved by the Russian Academy of Sciences. Because restrictions existed on the weight and volume of the habitat, two closely related species of snails were selected. Juvenile *H. aspersa* were used specifically for the histochemistry studies to provide an adequate sample size for each procedure. These snails are reared in laboratory under controlled conditions to allow selection based on age, weight, and quantity. For Foton M-2 and M-3 20 (1–4 g) and 20 (1–2 g) small *H. aspersa* were flown, respectively. A larger snail, *H. lucorum*, was desirable for the behavioral and electrophysiological studies. These snails were obtained through commercial vendors, and did not represent siblings or statistical cohorts except they were of relatively comparable weight within each group. For Foton M-2 and M-3 15 (12–18 g) and 16 (3–8 g) adult *H. lucorum* were flown, respectively.

Before flight all snails were weighed and an identifying number was painted on the shell of each large snail. The snail habitat was divided into 2 chambers to separately house the small and large snails. Snails are both hibernators (winter) and estivators (summer/dryness). Entering into either of these low metabolic states would conceivably mask any effect of the space flight. In an effort to prevent estivation, ample food and water sources were provided over the course of the mission, and the temperature within the habitat (and satellite) was maintained within 19–22°C (optimal temperature for the entire payload). Charcoal filters were also placed in the habitat to control fungal and algal growth. Inspection of the habitat upon return to the lab after landing of M-2 and M-3 clearly indicated that the snails were active and consumed the available food during the mission.

### Behavioral Studies

In the wild as the snail crawls along a plant its weight can cause the leaf and snail to drop vertically. The snail stays attached and faces head down, and will reorient itself by rotating 180° about its axis and crawl up the plant against the gravity vector. This behavior is termed negative gravitaxis. The negative gravitaxis response of each large snail was tested prior to launch and similarly tested postflight. In the test the snail was positioned on a horizontal plastic surface containing a fine grid pattern and hinged to permit a controlled 90° vertical pitch of the animal. The snail at this time, T0, is oriented with its long axis parallel to the vertical and head facing down. Typically, after a delay the animal re-orients itself by 180° and moves upward on the platform. Each test was recorded on video and the latency of re-orientation at 2–4 fixed body positions was calculated off-line. For M-2 mission all animals were tested in the negative gravitaxis paradigm. To allow the necessary time for the neural recording paradigms for the M-3 mission the behavioral testing was scored in 5 flight snails at T0–T2 latencies.

### Electrophysiology Studies

After the behavioral tests were concluded (M-2: 31–35 h after landing; M-3: 14–18 h after landing), the snail's nervous system was isolated and prepared for electrophysiological studies of the statocyst response to tilt mimicking the “head down” and the “tail down” behavioral conditions. To capture the likely neural response to the space mission it is critical that recordings are conducted not only as quickly as possible after the flight, but with the least amount of time between subjects. Thus we designed whole nerve recording techniques from the statocyst nerve, *n. vestibularis*, which contains the axons of 13 statoreceptors, and protocols to systematically and consistently record the greatest number of statoreceptors in the shortest period of time in each snail.

The preparation was made by rapidly cooling the snails to 4°C and anesthetized by injecting isotonic MgCl_2_ saline (∼15% of the animal weight). Next, the central ganglionic ring was dissected free from the animal and pinned to a silicone-elastomer (Sylgard)-coated dish. The tissue was bathed in a saline solution containing (in mM) 100 NaCl, 4 KCl, 7 CaCl_2_, 5 MgCl_2_, and 10 Tris-HCl buffer (pH 7.8). Neural commissures connecting the left and right pedal ganglia were severed. The dish contained two chambers that were isolated from each other using a Vaseline barrier. One chamber contained a pedal ganglion with an intact statocyst and the other contained the corresponding cerebral ganglion with attached ommatophores, and the two neural structures were connected by the *n. vestibularis* through the isolation bridge. In the M-2 experiments, the ganglia were first treated with Protease (1 mg/ml) (Type XIV, SIGMA, USA) for 10 min at room temperature, washed out with saline solution, and the connective tissue sheath was completely removed to improve recording quality. Only microdissection techniques were used in the whole nerve recordings of M-3 experiments to improve recording quality. Extracellular recording of *n. vestibularis* activity was made using Ag/AgCl electrodes, one placed in each saline bath of the two chambers. The signal was amplified (300–3000×) and filtered (20–2 kHz) by a differential amplifier (NB Lab, Russia), digitized (10 kHz conversion) and acquired into commercial software (Digidata 1200A A/D converter and Axoscope 9.0 software, Axon Instruments, USA), and stored on a computer for subsequent analysis.

**Figure 1:**
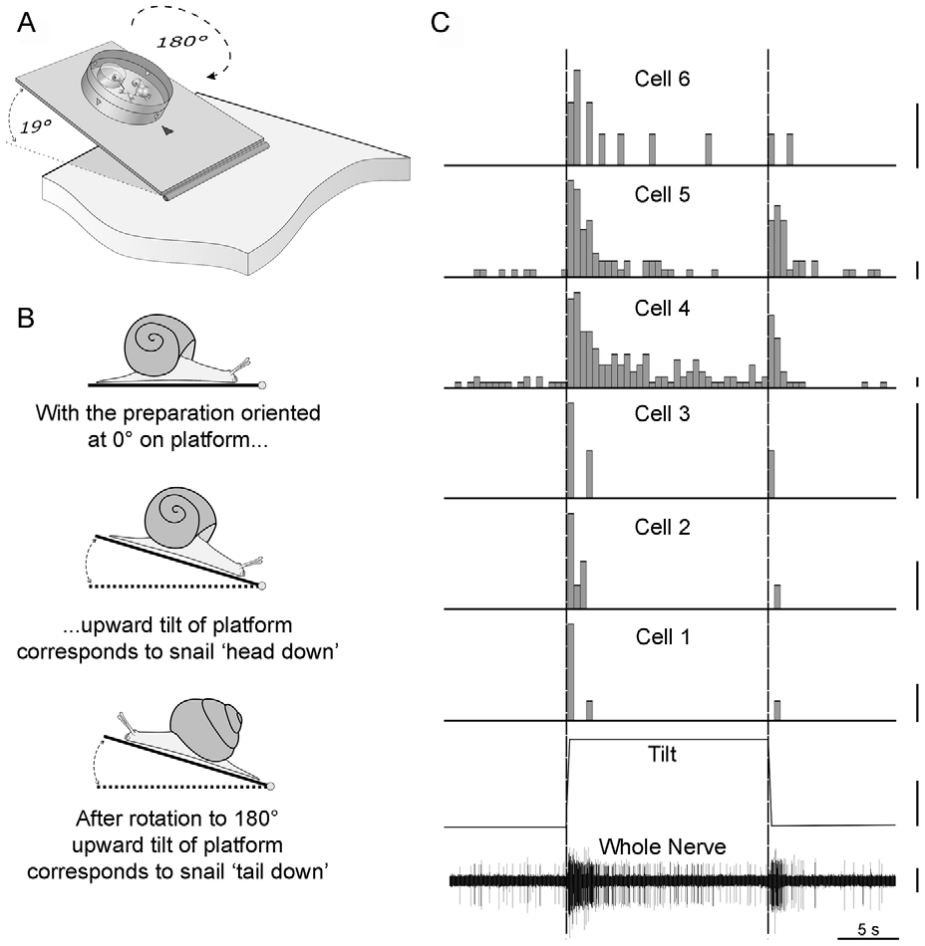
Experimental paradigm of behavioral and physiology tests. **A.** Cartoon of tilt platform used in neural recordings. Petri dish is fixed on a platform that can be mechanically tilted to a maximum displacement angle of 19°. In Foton M-2 experiments the tilt duration was fixed to 1.1 s (17.3°/s peak tilt velocity) and in Foton M-3 experiments the tilt duration was varied in 4 steps from 550–3020 ms (6.3–34.5°/s peak tilt velocity). Activity of the statocyst nerve was recorded using electrically separate chambers for statocyst and cerebral ganglion with the nerve passing over a Vaseline bridge. **B.** Head-down or head-up tilt of snail correspond to tilting platform with the preparation oriented at 0° (middle panel) and 180° (lower panel), respectively. **C.** Example of whole nerve response to tilt and cell sorting technique. Traces from bottom to top: whole nerve statocyst discharge (bar = 5 µV), platform position during tilt stimulus was recorded using a potentiometer (bar = 10°), and six identified cells in this preparation labeled Cell 1–6 (bar = 2 spikes/s).

**Figure 2:**
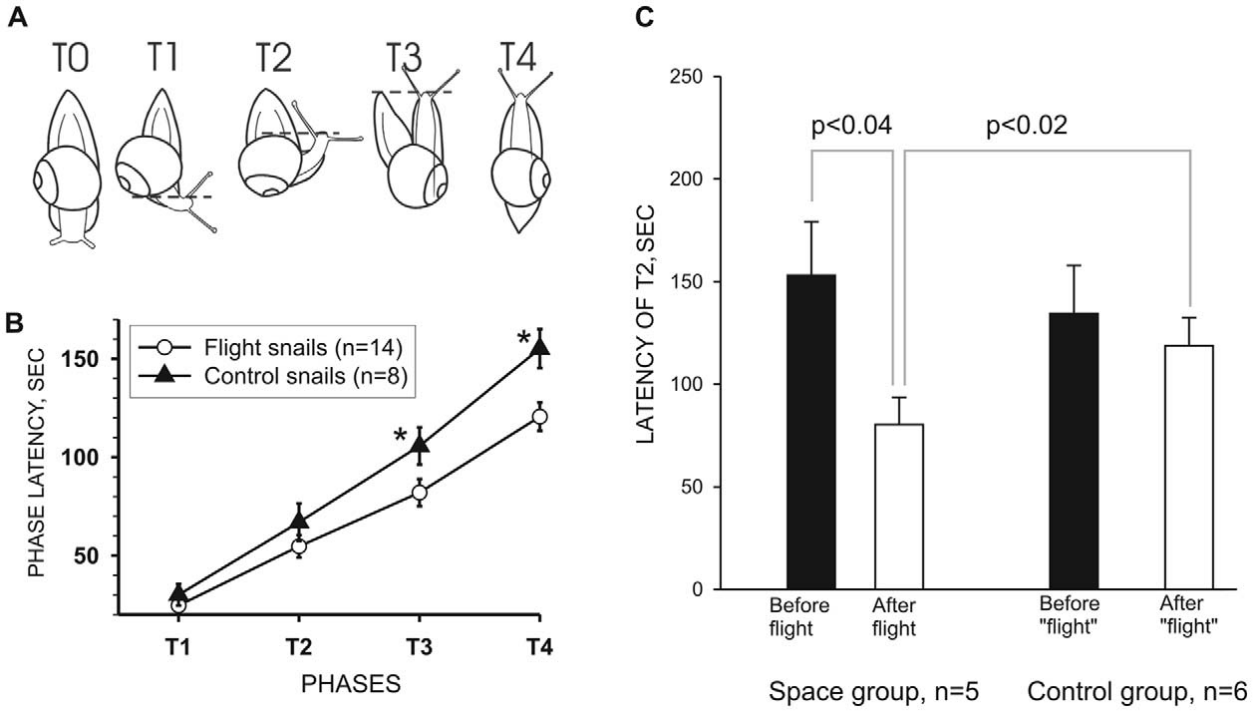
Negative gravitaxis response in control and postflight snails. **A.** Phases of the stereotypic response to sudden shift of the snail with platform from horizontal to “head down” position. **B.** Latency of gravitaxis reaction phases acquired during Foton M-2 experiments. The plot shows averaged (±SEM) time of the behavioral responses at 4 phases of the negative gravitaxis response in 14 flight and 8 control snails. Flight snails were faster in their response to pitch stimulation at each phase, and the difference reach level of significance p<0.05 at the later phases T3 and T4. **C.** Changes in latency of gravitaxis reaction of T2 phase acquired during Foton M-3 experiments. The plot shows averaged (±SEM) time of the behavioral responses at the T2 phase in 5 flight and 6 control snails tested before (black columns) and after (open columns) flight. Flight snails were faster than control snails as a group in their response to pitch stimulation, insignificant at T1 (not shown) but significant (p<0.02) at T2 phase. Post-flight gravitaxis responses were significantly faster (shorter latency of T2; p<0.04) than pre-flight responses recorded in the same snail.

Natural stimulation of the statocyst was performed on a custom tilt setup, which was slightly modified for the experiments of Foton M-3 to enhance testing. Figure 1A shows a cartoon of the tilt platform with the isolated neural preparation. In both series of experiments the preparation dish was positioned at 45° or 90° steps to test the statocyst's physiological response at different orientations with respect to gravity. 0° orientation was defined as the position in which rise of the platform (Fig. 1A) mimicked a downward pitch of the animal's head in intact snails (Fig. 1B, middle cartoon), and corresponded to T0 (see Fig. 2A) in the behavioral experiments. Increments in degrees were taken in a counter-clockwise direction, as viewed from above, up to a ‘tail down’ or ‘head-up’ snail tilt of 180° (Fig. 1B, lower cartoon). Platform tilt was pneumatically driven in M-2 experiments, and its direction was controlled by an electronically controlled valve manually switched on/off. To align the platform movements to the precise extracellular signal the start and finish times of tilt were manually tagged. Measured time of platform rise was 1.1 s (peak tilt velocity 17.3°/s), fall was 1 s, and the mid-point of tilt profile was set as time 0 for later analysis. We improved the stimulus paradigm for M-3 experiments by using a microcontroller-operated DC stepper motor with worm gearing to drive the platform tilt. The duration of tilt motion was thereby adjustable, and permitted delivering stimuli with different, but still brief, acceleration phases to ensure a response threshold to tilt was reached. This flexibility in adjusting the test protocol was particularly important in the postflight experiments where differences in threshold and response saturation might occur. We tested 4 different durations of 19° platform rise in the range from 550–3020 ms (6.3–34.5°/s). Angle of platform movement was measured by an in-line potentiometer, whose moving rod was used as the axis of rotation. The potentiometer measures were recorded on a separate channel and the beginning of movement was set as time 0.

Sample recordings and stimulations were first made to correct the volume of buffered saline, and the gain and filters settings. After this calibration procedure, the main protocol of stimulation started and was the same for all applied orientations. For Foton M-2 experiments the protocol was 1 min recording in the horizontal position, upward pitch to hold position and 30 s recording, followed by return to horizontal and 30 s recording, repeated 3-times with 30 s background recording between trials. For the experiments of Foton M-3 test epochs using different speeds of tilt were added that extended the main protocol to 1 min of recording in each position: horizontal — up (3020 ms ramp time)— down (3020 ms) — up (2085 ms ramp time) — down (2085 ms) — up (1075 ms ramp time) — down (1075 ms) — up (550 ms ramp time) — down (550 ms).

Spike sorting based on template extraction was used to identify the discharge pattern of individual statoreceptors. All records were imported into Spike2 (version5; CED, UK). Cursors were positioned to zero point of stimulation, marked by the tags (Foton M-2) or in accordance with auxiliary potentiometer channel (Foton M-3). Spike discrimination was made for all records in the built-in Spike2 module by template, made from every record. For individual records, different types of identified spikes varied from 2 to 12 (distinguished by non-intersecting principal component analysis clouds), probably depending on quality of nerve and statocyst preparation. Data of total nerve activity were analyzed by constructing post-stimulus histograms built on spike count per time bin (0.2 s for M-2 and 0.3 s for M-3) in the range of −10 to 30 s from zero point of tilt stimulation. For M-2 data the mean of 3 repetitive stimulations in main protocol was used for further analyses. An example of the whole nerve recording and spike sorting in one preparation is shown in Fig. 1C. Six individual cells were identified and their response properties are commonly encountered in all preparations. Statocyst cells have (Cells 4 and 5) or do not have (Cells 1–3 and 6) spontaneous firing rates, show a peak excitation during the transient phases of tilt (Cells 1–6), show a response decay following termination of the acceleration (and velocity) phases of tilt motion (Cells 1–6), and have variable levels of sustained excitation during the duration of the tilt displacement (Cells 4–6).

### Gene Expression Studies in the Snail Central Nervous System (CNS)

*In situ* hybridization (ISH) with digoxigenin-labeled RNA probes was used for preproHPep and preproFRMFa gene expression analysis in the CNS of control and postflight snails. The mRNA probe to preproHPep (Accession Number: U62544) was synthesized by D.A. Poteryaev of the Engelhardt Institute of Molecular Biology (Moscow) as previously described [11]. The mRNA probe to *Helix* preproFRMFa (Accession Number: L20768) was synthesized by F. Rozov of the Engelhardt Institute of Molecular Biology (Moscow) to 421 na. sequence of 3′ region of preproFRMFa cDNA. In control experiments no staining was detected with antisense probes to both peptides.

In Foton M-2 experiments the juvenile *H. aspersa* snails were divided into 2 groups for tracing the changes in preproHPep expression: the first half was prepared upon receipt of the snails at 30 h after landing and the other half was processed 12 h later. The test groups included 21 “naive” snails (10 naïve vivarium snails, 4 snails 24 h after foot cut, and 7 snails 24 h after 10 min exposure to 3.75 G centrifugation), 12 asynchronous control M-2 snails, and 12 postflight animals (6 prepared 30 h postflight and 6 prepared 42 h postflight).

In Foton M-3 experiments all *H. aspersa* snails were used for the ISH experiments and fixed upon receipt of the snails at 14 h after landing. Four *H. aspersa* and four *H. lucorum* were used for tracing the changes in FMRFa expression, and 16 *H. aspersa* for tracing the changes in preproHPep expression. Snails were anesthetized by injection of isotonic MgCl_2_ (∼15% of body weight). The central ganglionic ring was removed from the animal and pinned to a Sylgard-coated dish. After fixation for 2 h in 4% paraformaldehyde at room temperature (∼20°C), the connective tissue sheath was removed using fine forceps and scissors. Preparations were dehydrated and then rehydrated (to increase tissue permeability) by sequential incubation in 3∶1, 1∶1 and 1∶3 phosphate buffer-TWEEN (PTW)/methanol solutions. Preparations were treated next with 10 µg/ml Proteinase K solution, post-fixed in 4% paraformaldehyde, washed in glycine and PTW, treated with 1% hydroxylammonium chloride and prehybridized 6–8 h in hybridization buffer. Tissues were then moved to a hybridization buffer containing digoxigenin-labeled RNA probe and hybridized for 12–14 h. Preparations were then washed thoroughly, incubated in 10% heat-inactivated sheep serum and incubated in anti-digoxigenin antibodies conjugated to alkaline phosphatase for 12–14 h. Tissues were washed and incubated in substrate for alkaline phosphatase in the dark. The alkaline phosphatase substrate gives a deep-purple reaction product. Reaction was stopped after visual inspection with TRIS-EDTA buffer. Preparations were then cleared in xylene, mounted in Permount, and viewed under the light microscope.

### Immunochemistry

In the Foton M-2 mission we studied the changes in pattern of pedal peptide distribution in the nervous system and statocysts of *H. aspersa* by using whole-mount immunocytochemistry. The antiserum against pedal peptide (T**Pep**) was the kind gift of Dr. D. Willows (USA). Eight postflight animals and 11 control animals were used. Neural tissues were dissected in saline and fixed for 1 h at room temperature (RT) in 4% paraformaldehyde in phosphate-buffered saline (PBS). After fixation, the neural tissues were washed for 1 h in 70% ethanol, followed by several rinses in PBS. Prior to incubation with the primary antibody solution, whole-mounts were washed for 2 h in blocking solution containing 0.5% Triton X-100, 0.01% sodium azide, 5% normal goat serum (Sigma), and 1% bovine serum albumin (Sigma) in PBS. The staining procedure was done at RT using the blocking solution for all washes and antibody dilutions: primary antibody for 48–72 h followed by wash for 6 h; and secondary antibody for 24–36 h followed by wash for 6 h. Secondary antibody was AlexaFluor™ 488-conjugated goat anti-rabbit IgG (Molecular Probes, Caltag Laboratories) diluted to 1∶200. For archival storage, the whole-mount preparations were washed with PBS and embedded without dehydration series in Aqua Poly/Mount media (Polysciences, Inc). Preparations were examined using an AxioPlan (Zeiss, Germany) fluorescence microscope connected to a digital camera (Camedia C–4000; Olympus, USA) for acquiring images. In control experiments, the primary antiserum was omitted. No staining resulted in this series.

### Delayed Asynchronous Control Experiments

Two days after launch in both M-2 and M-3 missions a delayed asynchronous ground control test was initiated. Snails were housed in a comparable habitat and placed into a tightly sealed incubator whose temperature was adjusted according to the down-linked temperature data from the satellite, thereby exposing the snails to a contained atmospheric composition and to the same temperature profile as the flight snails. It should be noted that not all parameters between non-flight and flight snails are controllable. The major differences are as follows. Flight snails were transported to Baikonur Cosmodrome in Kazakhstan for launch. Snails were exposed to ∼9 min of varying acceleration (up to ∼9 G) before reaching orbit altitude. As the satellite re-enters Earth's atmosphere, the animals are again exposed to sustained and transient (e.g. parachute deployment) increases in gravitational forces until landing. After landing the satellite is opened, the contents are transported by helicopter to the nearest airfield, and flown to Moscow. In the case of Foton M-2 the delay was 30 h. For the M-3 mission a private charter was procured to get the specimens to the laboratory in 13 h.

## Results

### 1. Behavioral experiments

#### Foton M-2

Negative gravitaxis is a behavioral orientation response in the direction opposite to the gravity vector. To elicit this behavioral response the snail is pitched 90° head-down, akin to its own weight bending the leaf downward. The snail will detect this change in head orientation with respect to gravity, principally via the statocysts, and rotate its body 180° to face upward against the gravity vector. Flight snails in general re-oriented themselves faster than their control counterparts in this test. Cartoon in Fig. 2A shows the selected body positions where time measures were taken: at T0 the snail is pitched head-down from the horizontal plane, T1 is the time in which the snail has initiated a head turn, T4 is the final phase of complete re-orientation, and T2 and T3 are intermediate body positions in time (shown Fig. 2A). We scored the latency of each phase of behavioral performance from the videotape off-line. The plot in Fig. 2B compares the averaged latencies of the behavioral responses at the separate 4 phases for the 14 postflight and 8 control snails. Flight snails performed faster at each phase, and this was significant (p<0.05; nonparametric, two-tailed, unpaired Mann-Whitney test) at T3 and T4 (marked *).

#### Foton M-3

The protocol of behavioral testing of this mission was changed to accommodate the time required to complete the additional electrophysiology protocols. It was abbreviated to capture the initial fast re-orientation responses of phases 1 and 2 (T1 and T2 in Fig. 2A) following a horizontal to vertical head-down pitch of the snail at T0. We tested the latency of negative gravitaxis response before the flight in 6 control and 6 flight-designated snails and after the flight in the same snails (one flight snail died). Comparison of averaged latencies of the behavioral responses at the two phases for the negative gravitaxis response for the 5 flight and 6 control snails showed significant differences in performance. Flight snails reacted faster than their control counterparts to pitch stimulation (Fig. 2C), and this was significant (p <0.02; nonparametric, two-tailed, unpaired Mann-Whitney test), and faster after than before the flight in the same animal (p<0.04). Results clearly suggest the existence of changes in behavior of the postflight snails and confirm the results of Foton-M-2 mission.

### 2. Electrophysiological experiments in isolated CNS with intact statocysts

#### Background statocyst activity

Spontaneous firing rate of the statocyst nerve was measured in the dark in control and postflight snails in both Foton M-2 and M-3 experiments. Three 10 s intervals were taken for analysis in each record. Tilt stimulation was not presented for at least several minutes before the first analyzed interval. No significant (Mann-Whitney Rank Sum Test) difference in level of firing rate (average firing rate ± SEM, in spikes/s) was observed between control and postflight snails for both M-2 (n = 4) and M-3 (n = 8) missions: 5.6±1.03 vs 5.81±1.97 and 6.23±1.63 vs 5.73±0.92, respectively.

#### Summary of Statocyst Responses

**Figure 3:**
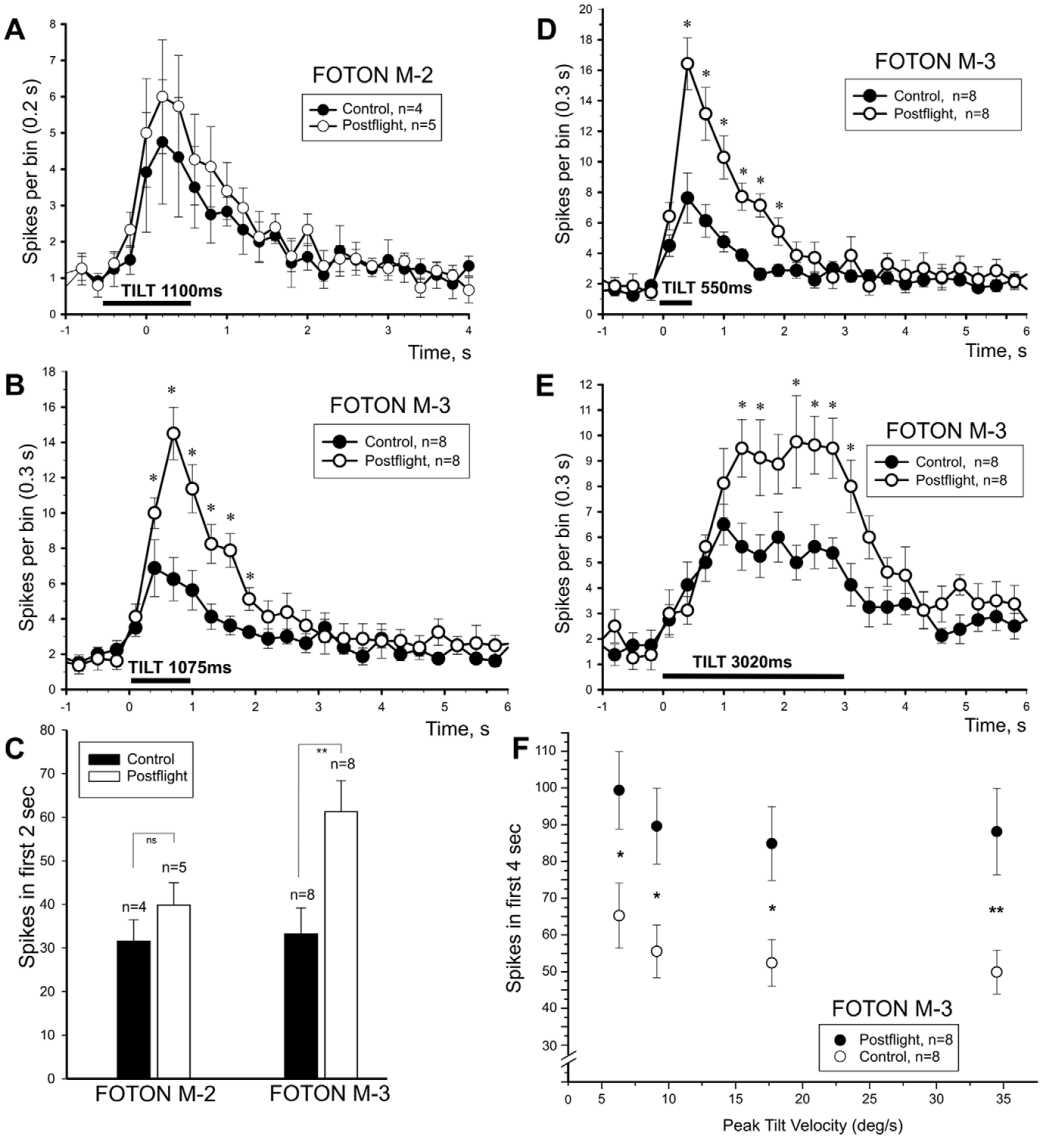
Postflight increase of statocyst response to vestibular stimulation. **A.** Averaged statocyst nerve responses (mean spike rate ± SEM sampled at 0.2 s bin width) of 5 postflight (open circles) and 4 control (filled circles) snails (Foton M-2) to platform tilt. The increased response of the postflight snails to tilt was insignificant. The stimulation and recording protocols were improved for the Foton M-3 experiments. Averaged statocyst nerve responses (mean spike rate ± SEM sampled at 0.3 s bin width) of 8 postflight (open circles) and 8 control (filled circles) snails to platform tilt of 1075 ms ramp time or 17.7°/s (**B**; close to M-2 ramp time), and at a faster (**D**; 550 ms or 34.5°/s) and a slower (**E**; 3020 ms or 6.3°/s) ramp times. At all tilt speeds the magnitude of the statocyst response was significantly increased (indicated by * in each plot) in postflight snails. **C.** Cumulative number of spikes over 2 s period following the onset of tilt for M-2 and M-3 experiments. Spike numbers were taken from time 0–2 s in the plots shown in panels A and B for control and postflight snails to allow a more direct comparison between the two missions. Control data were comparable in both missions, but the postflight results were significantly different in M-3 experiments (p<0.01, Student's t-test). **F**. The significant hypersensitivity of the statocyst to tilt following µG exposure is shown by plotting the total number of spikes (mean ± SEM) over a 4 s period following tilt onset at 4 peak velocities in the 8 control and 8 postflight snails (p<0.01**; p<0.02*).

We made significant improvements to the tilt apparatus for the M-3 experiments. These improvements impacted our ability and rationale to tabulate the responses of M-2 and M-3 experiments into one population, and thus they are presented separately. Table 1 gives the averaged cumulative responses of the control and flight snails in the 2 missions. Tilt responses were scored as the total sum of spikes during the first 2 s after onset of tilt. Data are derived from the records shown in Fig. 3A for M-2 and Fig. 3B for M-3, and displayed in the bar graph of Fig. 3C. An increase in magnitude of response to tilt after flight is evident in both sets of experiments, but it was insignificantly different from control records in the M-2 study. In the M-3 experiments, the difference in response magnitude between control and flight snails was the nearly two-fold and was highly statistically significant (p<0.01, unpaired Student's t-test).

**Table 1:** Averaged Cumulative Neural Responses to Tilt.

Snail	Mean ± SEM
M2 control	33.1±8.3 (n = 4)
M2 flight	42.2±8.7 (n = 5)
M3 control	33.3±5.9 (n = 8)
M3 flight	61.3±7.1 (n = 8)*

The total number of spikes was collected over a 2 s interval after the onset of tilt in control and postflight snails for both M-2 and M-3 experiments. The tilt duration was comparable for the M-2 (1100 ms; Fig. 3A) and M-3 (1075 ms; Fig. 3B) experiments. The control and flight snails had highly significant differences in magnitude of tilt response in the M-3 experiments (*p<0.01, Student's t- test for difference between the means).

#### Effect of tilt duration on statocyst response

The neural response of the statocyst to natural tilt stimulation was recorded in 5 flight and 4 control M-2 snails and in 8 flight and 8 control M-3 snails. The behavioral results of the snails of the same groups were obtained earlier or in parallel. The isolated neural preparations were placed in an orientation so that the rise of the platform corresponded to a head down tilt in the intact animal. Duration of tilt from one position to another for M-2 was fixed (Fig. 3A) and was 1 s (fall) to 1.1 s (rise, or 17.3°/s). In M-3 experiments the duration (speed) of rise/fall was varied in 4 steps from 550 ms (34.5°/s) to 3020 ms (6.3°/s). As a result the acceleration phase of tilt, although still brief, was varied to ensure a response threshold was reached in both the control and postflight snails. Responses are plotted in Fig. 3 at the fastest (D), slowest (E), and an intermediate speed (B) comparable to the M-2 tilt profile. It is evident from the results that in postflight animals the peak of response is significantly higher (p<0.01, marked by *, ANOVA with post hoc analysis) at all stimulus speeds, greater than 2-fold at the faster speeds (Fig. 3B and D) and nearly 2-fold at the slowest test speed (Fig. 3E). A similar, but not significant, tendency was observed after Foton M-2 flight (Fig. 3A). The neural population response to tilt is characterized by an initial rise in spike rate to a peak followed by decay, often exponentially, toward the baseline after tilt motion has stopped. This rate rise and fall was not altered by the space flight, only significantly elevated in postflight snails at each tilt speed (Fig. 3F; p<0.02*).

#### Effect of tilt direction on statocyst response

**Figure 4:**
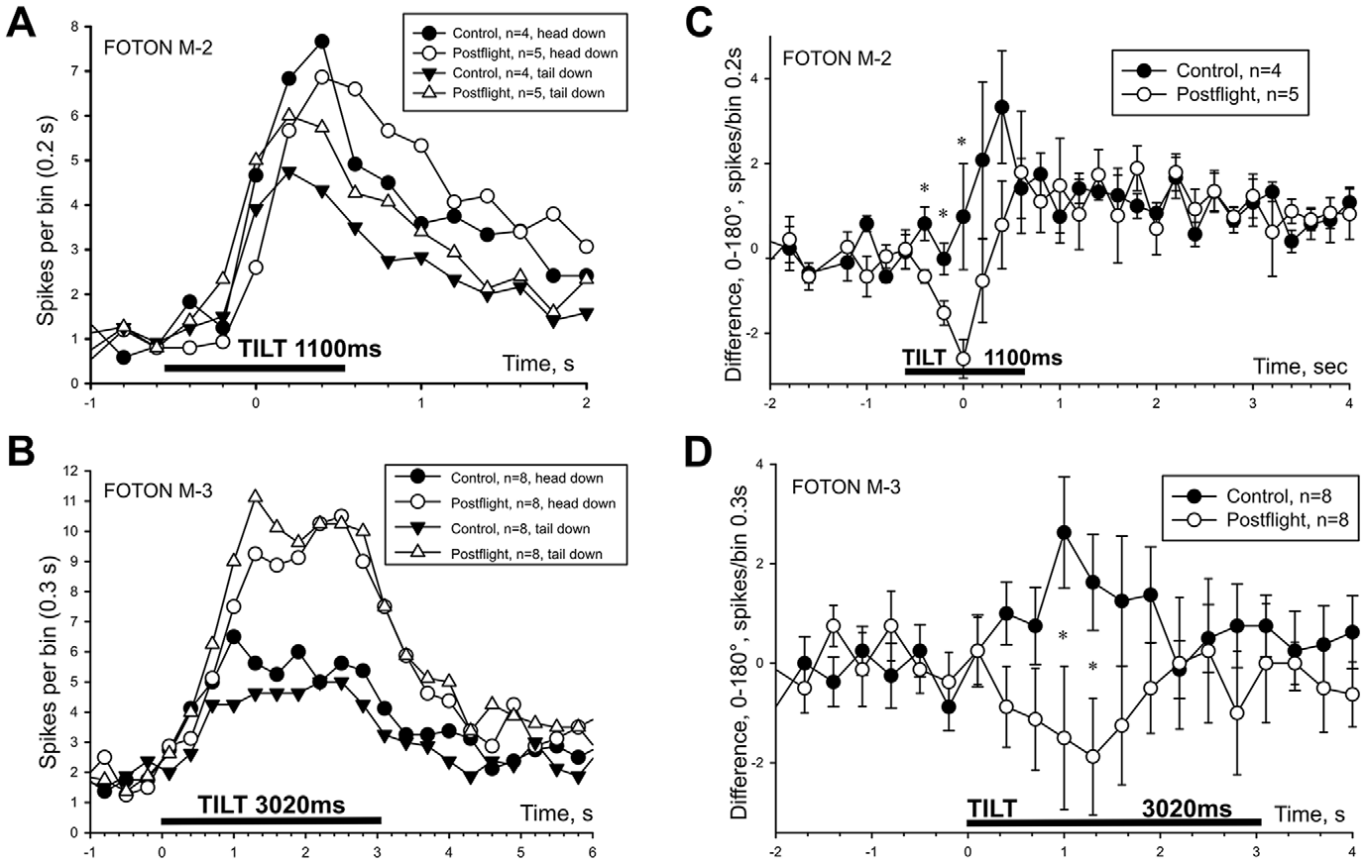
Postflight changes in directional sensitivity of statocyst response to tilt. **A, B:** Electrophysiological responses to tilt in statocyst nerve of 4 control and 5 postflight snails (**A**; Foton M-2) and 8 control and 8 postflight snails (**B**; Foton M-3) are shown at head down and head up (or tail down) orientations. Scales are expanded in each plot for illustrative purposes. **C, D**: averaged difference between statocyst nerve responses to tilt corresponding to “head-up” and “head-down” positions are plotted for M-2 (**C**) and M-3 (**D**) experiments. A response near zero indicates no directional preference. In both M-2 and M-3 series control and postflight snails the statocyst response demonstrated the opposite directional selectivity. A significant difference between postflight and control snails was observed in the middle portion of tilt in both M-2 and M-3 experiments (p<0.02, RM-ANOVA with Tukey post-hoc analysis).

In control animals the statocyst response to tilt at 0° orientation (corresponding to snail's head down) was consistently, but not significantly, larger than at 180° (snail's tail down) in both Foton M-2 (Fig. 4A) and M-3 (Fig. 4B) experiments. Responses at 90° and 270° orientations were similar to responses at 0° (data not shown). These results reflect an orientation selectivity of the animal that normally exists and is the basis of negative gravitaxis. But in postflight animals this directionality of statocyst responses to the same tilt stimulus was disturbed. To clearly illustrate this effect, we plotted a difference between the responses obtained at the two opposed orientations (Fig. 4C, D). To make this plot, the average number of spikes of the two responses were subtracted within each preparation and then averaged by group. If no preference to orientation exists, the difference of responses in the plot will be close to zero; and conversely the greater the distance from zero, the more significant is the difference. A significant difference between flight and control snail was observed at several time points for both M-2 (Fig. 4C) and M-3 (Fig. 4D) experiments indicating that responses differed for tilts in the opposite sense (statistical significance was evaluated by repeated measures ANOVA with Tukey post-hoc analysis, p<0.02 for several time points, marked by *). In control snails the statocyst is tuned to ‘head down’ tilt (and more spikes are generated at this orientation), while in postflight snails the directional selectivity was not so clear but tended to be opposite (Fig. 4 B, D). This finding suggests that the neural responses of the statocyst to adequate vestibular stimulation in the postflight snails are independent of the tilt direction, while in the control animals a directional preference for head down tilt occurs.

### 3. Postflight changes in gene expression in CNS

#### Foton M-2

The differential expression pattern of the preproHPep gene was examined in the CNS of postflight and asynchronous control snails, in centrifuged animals, and foot injury ground control snails. Snail CNS with statocysts was stained for products of expression of preproHPep gene: for proHPep mRNA using *in situ* hybridization (ISH) and for processed peptide using immunohistochemistry (IHC) with anti-TPep antibody. Stained cells containing the proHPep mRNA and HPep peptide were observed in cerebral, subesophageal ganglionic complex, and in pedal ganglia in all preparations. No systematic differences were observed between postflight and asynchronous control snails in general location and overall pattern of the stained ganglion neurons (data not shown). On the contrary, a qualitative difference in staining of the statocyst receptors was observed (Table 2). No staining was found in control snails, while in about 50% of statocysts a specific staining of 2–3 neurons adjacent to the vestibular nerve was observed in snails 30 h postflight. The number of stained statocyst receptors decreased after 42 h postflight. Naïve snails from the same batch showed no staining of statocyst receptors, whereas 24 h after a noxious stimulus was presented by an incision of the skin on the foot some staining was observed (Table 2). No significant differences were observed for pedal peptide expression pattern between IHC and ISH patterns of staining in CNS, both in control and flight animals. Nevertheless, the three statocyst receptors identifiable by their position were always stained by means of a specific antibody suggesting a high level of the neuropeptide in the normal state; and the same neurons were stained only in postflight animals using ISH suggesting an activation of transcription during µG exposure.

**Table 2:** Expression of the *Helix* Pedal Peptide (HPep) gene in statocysts of snails exposed to microgravity and under different ground conditions.

*Condition/Flight*	*Snails (N = )*	*Statocysts (N = )*	*Quantity of statocysts with HPep expression*
Naïve/M-2	10	20	No staining observed in statocysts
Naïve, 24 h after foot cut/M-2	4	8	2 stained neurons observed in 1 statocyst
Naïve, 24 h after 3.75 G centrifugation/M-2	7	14	No staining observed in statocysts
30 h postflight/M-2	6	12	6 statocysts (50%) showed staining
42 h postflight/M-2	6	12	4 statocysts (33%) showed staining
Control/M-2	12	24	No staining observed in statocysts
14 h postflight/M-3	16	32	Expression observed in 29 statocysts (91%)
Control/M-3	11	22	Expression observed in 13 statocysts (59%)

#### Foton M-3

In M-3 experiments we used only ISH for revealing postflight changes in mRNA expression of two genes, preproHPep and preproFMRFa. We examined the differential expression pattern of the preproFMRFa gene in control (n = 8) and postflight (n = 8) snails. The preproFMRFa gene was expressed in all circumesophageal ganglia, and the pattern of expression was very stable and similar in control and postflight preparations. No expression was found in statocysts of control or postflight snails. Thus, our results do not confirm the participation of this small neuropeptide in observed changes in behavior and statocyst function.

**Figure 5:**
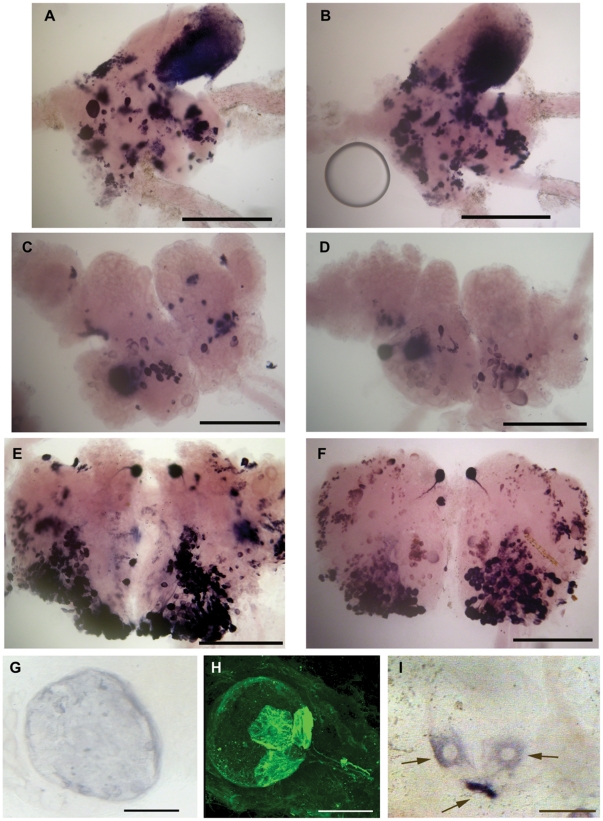
Localization of neurons expressing preproHPep gene in snail CNS and statocyst using *in situ* hybridization. Left panels (**A, C, E, G**) are images taken from control snails; right panels (**B, D, F, I**) are those taken from postflight snails. The staining in control and postflight snails was qualitatively similar in the CNS structures, but consistently different in the statocyst. **A, B**: cerebral ganglia; **C, D**: suboesophageal ganglia complex; **E, F**: pedal ganglia; **G, I**: statocysts. Note the labelled statocyst receptor cells in postflight snails in I (indicated by arrows) and lack of staining in control snails in G. **H**: for illustrative purposes the immunohistochemistry of HPep in a preflight snail shows the location of 3 receptors with respect to the statocyst nerve. Expression of this gene was observed only in these cells in all preparations. Calibration: A–F, 500 µm; G–I, 50 µm.

The expression pattern of the preproHPep gene was re-investigated in the postflight and control snails using ISH with mRNA following the Foton M-3 mission. The M-3 results qualitatively match those of the Foton M-2. Specifically stained cells were similarly observed in cerebral, subesophageal ganglia complex, and in pedal ganglia in all preparations. No systematic differences were observed between the postflight and control snails with respect to location and pattern of the stained ganglion neurons (Fig. 5, A–F). Once again a qualitative difference in staining of the statocyst neurons in postflight snails was observed (Fig. 5, G and I). In control animals (11 snails, 22 statocysts examined) the 3 specifically located neurons expressing preproHPep gene were found in 59% of cases, while in 16 postflight snails fixed 14 h after landing the expression was found in 96% of cases (Table 2). Since there are only 13 receptors in total in each statocyst, an up-regulation of gene expression in several of them represents possible changes in their function. This specific increase in gene expression in statocysts is indicative of the physiological load and may reflect the flight experience.

## Discussion

Space flight offers unique opportunities to study how gravity shapes life on Earth. It also offers novel challenges to the organism. Upon entry into microgravity (µG) the astronaut typically experiences a Space Adaptation Syndrome consisting of a varying degree of disorientation similar in many ways to terrestrial motion sickness. Within days symptoms mostly weaken and dissipate over the course of the mission. After space flight as brief as 1–2 weeks the astronaut undergoes a new adaptation phase or a re-adaptation to Earth's 1 G [12]. Vestibular studies conducted in space clearly show that the near elimination of the linear acceleration of gravity directly impacts the function of the human otolith organs [12], [13]. Studies in lower vertebrates, such as fish [7], [14], [15] and amphibians [16]–[19], have revealed remarkably similar results that reflect direct changes in gravireception of the otolith organs. The vertebrate otolith organs and the invertebrate statolith organs show a strong structural correspondence, likely due to their ancestral involvement in balance and equilibrium [20]–[22]. There is also developing evidence of molecular homology of the snail statoreceptors with the vertebrate hair cells [23]. Thus, it is possible that the snail might serve as a model organism in space neurobiology studies. In addition to Foton M-2 and M-3 (present study), snails have flown on the Shuttle, Russian space station MIR, and International Space Station missions in the past [24]–[27], and will be discussed in greater detail below.

In this study we investigated the structure and function of the gravi-sensing statocyst receptor as rapidly as possible following exposure to spaceflight in two separate missions. We assume that the observed changes were primarily the result of µG exposure. In-flight monitoring of statocyst function was not technically feasible in these missions. We observed a change, an upregulation, in the expression of a peptide associated with the statocyst. Pedal peptide was first identified by Lloyd in sea slug *Aplysia californica* [28]. The precise role of this peptide in the nervous system is still unknown. However, it has been shown that pedal peptide modulated the ciliary beat frequency of epithelial cells in *Tritonia* [29]. In 1997 the gene encoding pedal peptide was identified in terrestrial snail *Helix lucorum* [11]. Based on *in situ* hybridization data this gene is widely expressed in the nervous system of *Helix* and in some statoreceptors as well. Taking into account the known effect of pedal peptide on cilia beating and muscle contraction [30], we can assume that it might play an important role in the regulation of mechanical transduction processes of the hair bundles of statoreceptors, such as regulation of actin-myosin complex within the cilia [31]–[33], and the observed change in expression of gene encoding pedal peptide (Table 2) suggests existence of lasting influence of the flight.

Despite the small number of receptor cells in each statocyst, they were uniformly labelled for this pedal neuropeptide. A feasible interpretation of these HPep data is the existence of a mosaic expression of different peptides in the receptors [8]. This mosaic pattern is believed to encode varying information on the position and relative movement of the snail. In this case the degree of HPep expression from an almost negligible level to a significant level may indicate a change in signal transmitted to the nervous system from the gravi-sensing organ. It is tempting to speculate that the HPep expression patterns observed in postflight snails directly reflect, at least in part, the recorded behavioral and physiological changes, but this remains to be proven.

In the M-3 mission we also investigated the expression pattern of the FMRFamide gene in postflight and control snails using mRNA expression techniques. Although FMRFamide is not normally expressed in statocyst receptor cells [34], this neuropeptide can act to reduce calcium influx via regulation of the cell's secretory machinery [35], thereby modulating synaptic transmission, and perhaps playing a role in synaptic plasticity [36]. More recent evidence suggests that the expression and regulation of an FMRFamide-related neuropeptide gene family are possibly linked to the state of the organism [37]. No differential expression of the preproFMRFa gene was observed in the statocysts of control or postflight M-3 snails. Thus, our data suggest that FMRFamide is an unlikely candidate involved in the statocyst response to the novel space environment.

We measured the whole statocyst nerve activity in one recording session in control and postflight snails, and observed a significant increase in the total number of spikes in the postflight snails. This technique minimized any potential trauma to the statocyst organ itself and was considered the optimal method given the time-sensitive nature of the data. However, it does leave open the question as to the source of the overall level of excitation. The postflight increase in the total spike number could be the result of amplifying the normal discharges of responding cells or recruiting otherwise non-responding cells or both. To adequately answer this question we must wait until preflight and postflight recordings are made in each animal under identical recording conditions.

The physiological results reported here in the isolated statocyst in snails are in line with the vertebrate data [7], [12], [13], and conform to the proposition that µG exposure leads to changes in gravireceptor function. At the same time this similarity in neural response to µG exposure between the vertebrates and invertebrates is intriguing: the increased neural sensitivity in the vertebrate was detected in the otolith nerve afferents [7], one synapse away from the hair cell, whereas the increased neural sensitivity reported here in the snail was detected directly at the receptor level. Both the vertebrate otolith and invertebrate statolith structures are physically arranged for an optimal response to 1 G perceived during their development, and any departure from this arrangement in either direction will induce a perturbation of the detection mechanisms. However, the common neural response might be driven by shared or completely independent mechanism(s) with reference to adaptation to altered gravity. Different speculative hypotheses have been proposed [7]. In light of the current results in snails we need to re-open this debate. Possible mechanisms underlying the adaptive changes in statocyst function following µG exposure might involve a structural reformation, a change in neural signaling, or even a mixture of both.

### Structural Changes

A widely considered mechanism is a change in the weight-lending statoconia (or otoconia). Structurally, these are minute calcareous particles surmounting the neural epithelium. In µG, it is argued, the organism counters the loss of the gravity vector by increasing calcium carbonate production and depositing more mass on the statoconia. The total weight of the statoconia mass remains negligible while in µG, and thus changes are seen upon return to normal 1 G. A number of studies support this argument. The test mass (utricular or saccular otolith or statolith) of the fresh-water pond snail, *Biomphalaria glabrata* [25], marine mollusk, *Aplysia californica* [24], *Xenopus* [38], [39], newt [24], and swordtail fish [25] reared in space increased relative to ground-reared controls. In the opposite sense the test mass in *Aplysia californica* [24] and cichlid fish [25] born in hypergravity was reduced [24]. Exposure to µG has been linked to an increased growth of the statoconia in *Helix* [26], similar to the reported results in other species.

However, the predominant evidence in other species studied during developmental or adult stages is either no effect on test mass or ambiguous results [40]–[47]. In the fish the neural response to µG exposure was manifested in hypersensitivity without a distortion of its directional selectivity, and readaptation, defined as the process of adaptation to normal gravity, occurred relatively quickly [7]. In the present results the snail's neural response to µG exposure was manifested also in hypersensitivity, but with a loss of its directional selectivity. Although this finding does not confirm a structural change has occurred in the snail, it cannot be ruled out. It may also be hypothesized that the unweighting of the statoconia mass in µG induces a change in the mechanical linkage between the statoconia and hair cell stereocilia, but a morphological analysis is required to test this possibility.

The prevailing evidence in the vertebrate pointed towards an increase in synaptic strength originating in the hair cell and conveyed to the afferent contacts. The experimental findings in rat [48], [49] provided direct evidence that reformation of the hair cell synaptic machinery can be caused by µG. An increase in the number of synaptic ribbons in rat utricular hair cells as a result of µG exposure is in line with the increased sensitivity found in fish utricular afferents [7]. In the snail (present results) the increased sensitivity was found directly in the hair cell itself, thus excluding a mechanism utilizing plasticity of the hair cell-afferent synaptic complex. It should be noted that no direct membrane measurements have been taken from vertebrate hair cells following µG exposure, thereby leaving open the possibility that the increased sensitivity observed in the nerve afferents simply reflected an increased sensitivity of the receptor potential in the hair cell itself. In other words, a common mechanism in regulation of the hair cell sensitivity between invertebrate and vertebrates cannot be excluded.

### Changes in Cellular Signaling

A direct action on intracellular signaling might occur in the gravi-receptor itself. Without empirical evidence acquired in flight we can only speculate on the possibility of modifiable elements, such as changes in myosin motors or ionic channels, within the cell. Sensory receptors in invertebrates can generate action potentials and these potentials are modifiable by changes in environmental conditions, for example during learning. The primary sensory neuron is the first site at which cellular plasticity occurs during classical conditioning [50]. In the conditioned *Hermissenda* an enhanced excitability in type B photoreceptors involves a reduction in the peak amplitude of voltage-dependent (I_A_, I_Ca_) and Ca^2+^-dependent (I_K,Ca_) currents, an increase in the input resistance, and an increase of firing rate elicited by the conditioned stimulus (CS) or extrinsic current [51]–[53]. In type A photoreceptors an enhanced excitability to extrinsic current, increases in CS-elicited firing rate, and decreases in the magnitude of two K+ currents are seen [54]–[58]. In *Lymnaea* classical conditioning can be directly mediated by the statocyst hair cells and sensory information for associative learning converges onto the cell [59]. At least 3 distinct K+ currents are expressed in the soma of hair cells [60] and modification of these channels would likely influence its excitability.

Mature vertebrate hair cells develop generator or receptor potentials, but their plasticity to environmental variations is largely unknown. Delayed expressions of voltage-gated sodium current [61] and potassium currents [62] have been reported in neonatal utricular hair cells of rats raised in hypergravity, suggesting a direct influence of altered gravity on sensory cell sensitivity. Prolonged deviation of the sensory hair bundle can lead to adaptation of otolith hair cell receptor potentials [63], [64]. In the opposite sense prolonged unweighting of the otolith mass and thus tonic unloading of the otolith stereocilia could conceivably lead to adaptation of receptor potentials. A manifestation of this adaptation can be seen in the responses of some otolith afferents to prolonged steps of linear acceleration [65], [66]. Microgravity might be thought of as a step of motion of the hair bundle in the “off” direction for the hair cell, and adaptation in the transducer mechanism might negate any aberrant afferent response in µG. The onset and time course of adaptation response of the transducer might be both rapid and long-lasting, but remains conjectural.

One last mechanism needs to be considered. In addition to the feed-forward flow of signals from the hair cells to the more central cell areas that process the information to provide orientation, balance, and muscle tonus, a centrifugal pathway exists from the more central areas back to the sensory organs themselves. This efferent pathway is known for the gastropod statocyst [67] and cephalopod [68], [69], and in vertebrates for all hair cell structures [70]–[73]. In *Hermissenda* hair cells are capable of inhibiting neighboring hair cells and those on the opposite side via chemical and electrical synapses [74], [75]. In the toadfish efferent vestibular neurons directly inhibit the hair cell by shunting its membrane potential and at the same time increase the afferent firing rate [76], thereby effectively enhancing signal processing, for example, during large and rapid escape or feeding behaviors. In series of experiments on a Foton satellite and aboard the Mir space station this feedback mechanism was studied in the snail [27]. Eliminating the feedback to the statoreceptors by severing its nerve between 31–36 h after landing did not alter the firing rate in proximal portion of the nerve in postflight snails, but it did so in control snails, leading these investigators to conclude that µG exposure might inactivate the efferent innervation of the statoreceptors. Although it is likely that hair cells are under a continuous efferent adjustment based on prevailing behavioral requirements, it is not yet known whether the efferent innervation of the hair cell organs in any species is capable of a long-term calibrating influence based on the existing gravity level.
